# Acoustic Emission Monitoring of Carbon Fibre Reinforced Composites with Embedded Sensors for In-Situ Damage Identification

**DOI:** 10.3390/s21206926

**Published:** 2021-10-19

**Authors:** Arnaud Huijer, Christos Kassapoglou, Lotfollah Pahlavan

**Affiliations:** 1 Department of Maritime and Transport Technology, Delft University of Technology, 2628CD Delft, The Netherlands; L.Pahlavan@tudelft.nl; 2 Department of Aerospace Structures and Materials, Delft University of Technology, 2628CD Delft, The Netherlands; C.Kassapoglou@tudelft.nl

**Keywords:** structural health monitoring, acoustic emissions, piezoelectric wafer active sensor, embedded sensors, carbon fibre reinforced plastic, waveform similarity

## Abstract

Piezoelectric sensors can be embedded in carbon fibre-reinforced plastics (CFRP) for continuous measurement of acoustic emissions (AE) without the sensor being exposed or disrupting hydro- or aerodynamics. Insights into the sensitivity of the embedded sensor are essential for accurate identification of AE sources. Embedded sensors are considered to evoke additional modes of degradation into the composite laminate, accompanied by additional AE. Hence, to monitor CFRPs with embedded sensors, identification of this type of AE is of interest. This study (i) assesses experimentally the performance of embedded sensors for AE measurements, and (ii) investigates AE that emanates from embedded sensor-related degradation. CFRP specimens have been manufactured with and without embedded sensors and tested under four-point bending. AE signals have been recorded by the embedded sensor and two reference surface-bonded sensors. Sensitivity of the embedded sensor has been assessed by comparing centroid frequencies of AE measured using two sizes of embedded sensors. For identification of embedded sensor-induced AE, a hierarchical clustering approach has been implemented based on waveform similarity. It has been confirmed that both types of embedded sensors (7 mm and 20 mm diameter) can measure AE during specimen degradation and final failure. The 7 mm sensor showed higher sensitivity in the 350–450 kHz frequency range. The 20 mm sensor and the reference surface-bounded sensors predominately featured high sensitivity in ranges of 200–300 kHz and 150–350 kHz, respectively. The clustering procedure revealed a type of AE that seems unique to the region of the embedded sensor when under combined in-plane tension and out-of-plane shear stress.

## 1. Introduction

The layered nature of fibre-reinforced plastic (FRP) materials allows the incorporation of sensors, such as piezoelectric wafer active sensors (PWAS) [1] or fibre-Bragg gratings (FBGs) [2]. Placing sensors within the structure gives the possibility of monitoring the structure continuously without adversely affecting the hydro- or aerodynamics nor having the sensors exposed to harsh environments [3]. From the 1980’s [4] to present, a multitude of applications for embedded PWAS were proposed and investigated, ranging from strain measurement [5,6], energy harvesting [7], vibration control [8], and excitation and measurement of guided waves [9,10,11,12], measurement of electromechanical impedance [13] and acoustic emissions (AE) [14,15]. The latter is the focus of the present paper for application to structural health monitoring (SHM).

Functioning of the embedded PWAS can be assessed through static capacitance measurements [16,17], impedance measurement [17] and Hsu-Nielsen tests [14]. The measurement of AE, when using piezoelectric sensors, is influenced by the spectral characteristics of the sensor [18]. This influence may skew interpretations on whether certain damage mechanisms are occurring, or on the frequency content that a certain damage mechanism may emit. For embedded PWAS, various models to simulate sensor behaviour exist [19,20,21]. Nonetheless experimental assessment of the performance and sensitivity of the sensors for AE measurement has not been sufficiently researched and needs further development.

Embedding PWAS in a composite structure may also influence structural integrity and possibly, damage mechanisms [10,12,16,22,23]. Ghezzo et al. [24,25] compared specimens with and without an embedded device. AE in GFRP specimens loaded under tension with embedded devices were observed to have higher peak frequencies (up to 350 kHz) compared to baseline specimens (up to 180 kHz, depending on lay-up). Such signals were attributed to debonding and matrix cracking at the embedded device location. Xiao et al. [26] noted significant effects in AE energy and cumulative energy between CFRP specimens with and without embedded devices when loaded under tension. Early high-energy signals from the specimens with the embedded device were ascribed to CFRP-device interface delamination. This interface delamination is mentioned to cause further AE to be of relatively low energy, as compared to the energy of AE captured in baseline specimens. At the failure stage, lack of differences in the AE energy between specimens with and without embedded devices is associated with mutually occurring failure modes, such as fibre-breakage. The studies mentioned present case-specific results and it is uncertain to what extent the conclusions are applicable in situations with different loading conditions or different embedded devices. Further a method for clustering of AE signals in specimens with and without embedded sensors can provide a rigorous basis for assessment of observations, however to date it does not seem to have been reported.

Classification methods have been employed to relate AE to damage mechanisms in baseline FRPs [27]. Typically these methods rely on seeking similarities in certain key features of the AE, such as signal rise time, energy, amplitude, counts and duration in the case of Masmoudi et al. [14] and rise angle and average frequency in the case of Friedrich et al. [28]. In other research, in conjunction with assessment of aforementioned key features, the full waveforms are used [29]. For characterisation of damage in reinforced concrete structures, Kurz [30] and van Steen et al. [31,32] proposed clustering via similarity matrices. In such an approach, all waveforms are compared to each other using cross-correlation. Highly correlated waveforms are then clustered together. This method is well established in seismics to identify specific seismic emissions [33] and is expected to be greatly useful in other fields as well.

This research is aimed at providing insight into (i) qualitative assessment of the response of embedded piezoelectric sensors to damage-induced AE in CFRPs, and (ii) identification of clusters of AE that can be related to the possible damage induced by sensor embedment. To be able to assess these, CFRP beam specimens are manufactured by prepreg materials in three categories: no PWAS, smaller PWAS embedded, and larger PWAS embedded. Experimental sensitivity assessment of the embedded sensor mainly revolves around mirroring the response of the small PWAS to that of the large PWAS during four-point bending of CFRP specimens up to failure. Concurrently, surface-mounted sensors monitored the AE behaviour in all specimens. A clustering method based on waveform similarity is employed to discern AE that can be related to a damage mechanism related to the embedment of PWAS.

The manufacture of the specimens, the experimental procedure, and the clustering method are explained in Section 2. Results are presented in Section 3, including observations related to the characterisation of the PWAS and findings from the AE waveform clustering. Further discussions and conclusions are presented in Section 4.

## 2. Materials and Methods

### 2.1. Experimental Procedure

#### 2.1.1. Manufacturing of Specimens

**Figure 1:**
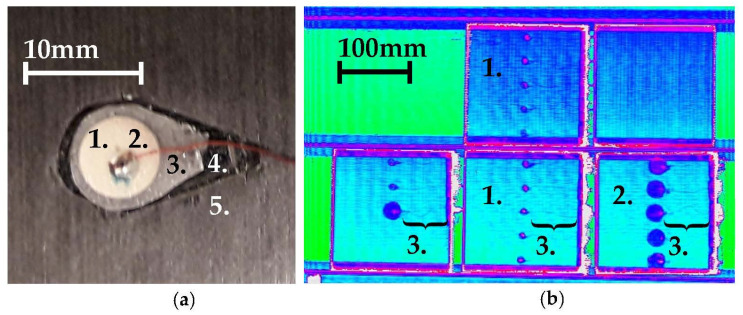
Manufacture of the specimens: (**a**) Close-up view of a ‘small’ sensor being embedded. Numbers 1 to 5 relate to the PWAS, solder and wiring, GFRP insulation, CFRP cut out and CFRP host material respectively. (**b**) C-scan after curing of CFRP panels with embedded sensors. Specimens were cut to width afterwards. Numbers 1 to 3 refer to panels with small embedded sensors, panels with larger embedded sensors and embedded wiring.

The specimens under investigation are coupons made from AS4/8552 unidirectional prepreg CFRP panels stacked in a [[0°, 90°]_7_,0°]_s_ symmetrical cross-ply lay-up. On predefined locations along the midline of the panel (in the length and width directions) and from the 21st to 25th laminae, drop-shaped cut-outs were made to accommodate the PWAS. The sensor lead wire was embedded between the 22nd and 23rd laminae. A close-up view of the 22nd lamina including embedded sensor is given in Figure 1a. After embedding the sensor system and placing the top laminae, the CFRP laminate was debulked and autoclave-cured following the procedure recommended by the supplier [34]. A C-scan of the cured panels is shown in Figure 1b. After curing, the panels, with a thickness of 5.4 ± 0.1 mm, were cut into coupons of 150 mm length and 27 mm width using a Proth diamond saw.

The PWAS is made of PZ27 soft composite PZT material [35] poled in thickness direction with silver electrodes on the top and bottom surfaces. This material has a recommended maximum temperature of 250 °C, which is 70 °C higher than the CFRP curing temperature. Two sizes of PWAS are considered; a ‘large’ type with a 20 mm diameter and 0.29 mm thickness, and a ‘small’ type, with 7 mm diameter and 0.24 mm thickness.

Sensor lead wiring, made from bifilar urethane-enamelled copper with a 0.15 mm diameter, was soldered to the PWAS using silver solder (S-Sn95Ag4Cu1). To prevent the wiring from getting damaged at the edge of the laminate due to the prepreg curing procedure, SMC style connectors were located at the laminate edge.

The conductivity of CFRP demands the PWAS and connector to be electrically insulated. The PWAS is laminated between two single layers of woven GFRP (HexForce 00106 with Araldite LY5052) resulting in a total sensor thickness varying between 0.7 mm and 1.2 mm. After curing, the sensor assembly was trimmed to shape. No processing of the GFRP surface has taken place. The connector was placed at one of the specimen ends during embedding between two layers of polyimide tape. Prior to and after embedding, the static capacitance of the sensor was evaluated to assess its post-manufacturing integrity.

In total 25 specimens were manufactured, among in which 18 PWAS were embedded.

#### 2.1.2. Measurement Procedure

**Figure 2:**
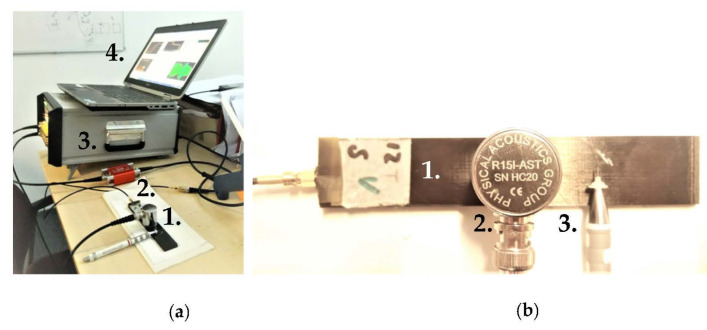
Set-up for exciting and measuring a Hsu-Nielsen source [36]: (**a**) Overview of the set-up, with the numbers 1 to 4 corresponding to the specimen, AEP-5H preamplifier, AMSY6 data acquisition system and a measurement computer. (**b**) Close-up showing the specimen (1.), AE sensor R15I-AST (2.) and a mechanical pencil with Nielsen shoe (3.). Note that in the final assessment a pencil with 0.5 mm diameter lead was used at a distance of 60 mm from the centre of the sensors.

**Figure 3:**
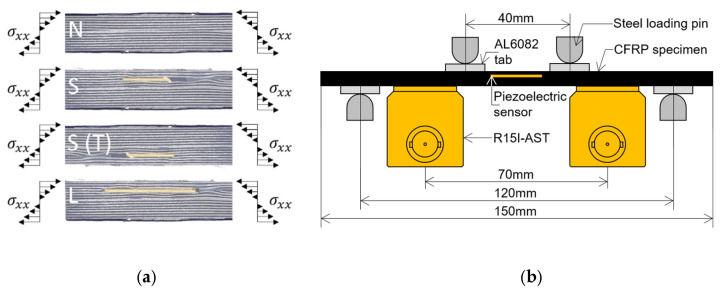
Four point bending experiment. In (**a**) the different types of specimens that are tested are visualised, with specimen abbreviation, sensor location and loading condition. (**b**) shows the dimensions and items involved in performing the four-point bending test.

**Figure 4:**
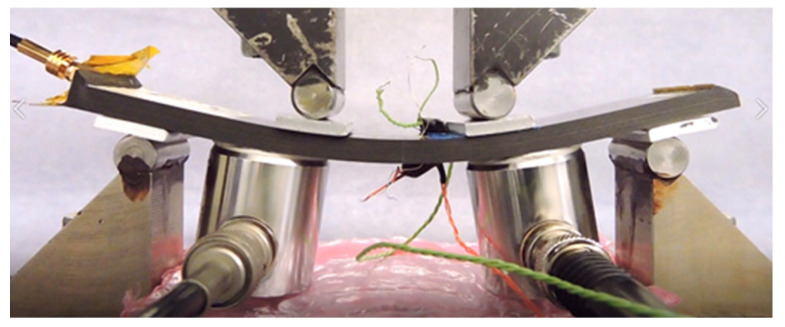
Specimen L3 with large embedded sensor in the four-point bending set-up close to failure.

Non-destructive and destructive tests have been carried out to assess the sensitivity and performance of the embedded sensor in AE measurement of damage in CFRP laminate. In a preliminary non-destructive test, the performance of the embedded PWAS is assessed by exciting the specimen at least five times with a Hsu-Nielsen source at a distance of 60 mm from the specimen centreline using a mechanical pencil with 0.5 mm H lead. At the top centre of the specimen, a reference R15I-AST sensor was also mounted using adhesive putty as couplant. The setup can be seen in Figure 2. In the destructive flexural test, two R15I-AST were placed at the bottom of the specimen, as shown in Figure 3b and Figure 4 the PWAS response is amplified with a 40 dB AEPH5 preamplifier, while the R15I-AST sensors had a 40 dB built-in preamplifier. Data was acquired using a Vallen AMSY6 system, which is able to record the full waveform based on hit definition. A digital filter was applied, allowing measurement only between 20 kHz and 960 kHz. Amplitude thresholds were set at 40 dB in all cases, with the exception for one embedded PWAS where a 70 dB threshold was used due to the experienced high background noise. The full waveforms were recorded with a sample frequency of 10 MHz, which in the analysis was resampled to 2.5 MHz. A 200 μs pretrigger time is used to get an indication of the signal-to-noise ratio as well as to allow appropriate time picking for low-amplitude signals. The measurement length of the waveform was adaptive and ranged between 250 μs and 1000 μs. A rearm time (hit lockout time) and duration discrimination time (hit definition time) of both 250 μs were applied. No peak definition time was specified. Hit definition settings were identical for embedded and surface-mounted sensors.

In the destructive tests, four types of specimens were subject to a four-point bending loading. Next to four baseline specimens (named N), two specimens with small embedded PWAS on the compressively loaded side of bending set-up (S), two specimens with large embedded PWAS loaded on the compressive side (L), as well as two specimens with small embedded PWAS placed on the tensile side of the bending setup (S_T_). The different specimen types are visualised in Figure 3a. Further on one L-type and two of each S and S_T_ specimens were tested to determine the ability of the embedded PWAS to measure damage-related AE. The embedded sensor is placed in the centre of the specimens, facilitating that the four-point bending creates a uniform strain field over the sensor region. A schematic of the four-point bending set-up can be seen in Figure 4. The set-up consists of four steel loading pins of 10 mm diameter that are placed within a Zwick/Roell 20 kN universal testing machine. To prevent the loading pins from crushing the CFRP, AL6082 loading tabs with 3 mm thickness and 15 mm width were placed between the CFRP and loading pins. In the second stage of experiments, the loading tabs were electrically insulated using polyester film tape to avert interference between the sensor, CFRP and test set-up. A loading force $F_{LC}$ was monotonically applied with a crosshead velocity of 1 mm/min. The experiment was halted when the specimen had failed, i.e., when instantaneous $F_{LC}$ dropped to half or less of the maximum $F_{LC}$ measured. A picture of specimen L3 with a large embedded PWAS close to final failure can be seen in Figure 4.

### 2.2. Clustering Procedure

To relate AE waveforms to specific degradation modes, a hierarchical clustering approach employing similarity measures is used, as described by Van Steen et. al. [31]. Waveforms recorded during the testing of specimens were compared to each other using normalised cross-correlation. For a pair of waveforms f(τ) and g(τ), as function of time (τ), the cross-correlation is defined as:

(1) $$\left(f⋆g\right)\left(t\right)=\overset{+\infty }{\underset{-\infty }{\int }}f\left(\tau \right)g\left(\tau -t\right)d\tau$$

Here, t represents a time shift between the waveforms. The resulting signal is normalised with respect to the autocorrelations of the two initial waveforms (Equation (2)).

(2) $$\bar{R_{fg}}\left(t\right)=\frac{\left(f⋆g\right)\left(t\right)}{\sqrt{\left(f⋆f\right)\left(0\right)\left(g⋆g\right)\left(0\right)}}\;$$

Waveforms should be of equal length in this procedure. This is ensured by imposing a maximum signal length of 250 μs or if shorter, the length of the shorter signal. The start of the waveform is defined by the first minimum of the Akaike Information Criterion (AIC) [37,38]. In Equation (3), the AIC varies over signal index n, and is dependent on the signal up to n ($U_{n-}$), the signal after n ($U_{n+}$) and signal length N [39].

(3) $$AIC_{n}=n\log_{10}var\left(U_{n-}\right)+\left(N-n-1\right)\log_{10}var\left(U_{n+}\right)$$

The normalisation from Equation (2) yields a cross-correlation parameter $\bar{R_{fg}}\left(t\right)$ varying over time shift with a value between −1 and 1. The maximum value may be seen as a measure of similarity, or similarity index (Equation (4)), between the waveforms, with 1 and −1 indicating a perfect replication of the waveforms of the same or opposite sign while 0 is indicative for two very dissimilar waveforms.

(4) $$similarity=1-dissimilarity=\max\left(\bar{R_{fg}}\left(t\right)\right)\;$$

By comparing all waveforms to each other, a matrix is acquired containing the similarity indices. Waveforms are grouped through average linkage, using the difference in mutual dissimilarity indices as a measure of inter-cluster distance [31]. The result is a dendrogram with similar waveforms sorted and linked to other waveforms at the value of their shared dissimilarity. Clusters are obtained by setting a threshold to the dissimilarity value, thereby dividing a group of waveforms from another group of waveforms.

## 3. Results

### 3.1. Measuring AE with Embedded Sensors

#### 3.1.1. Preliminary Testing

**Figure 5:**
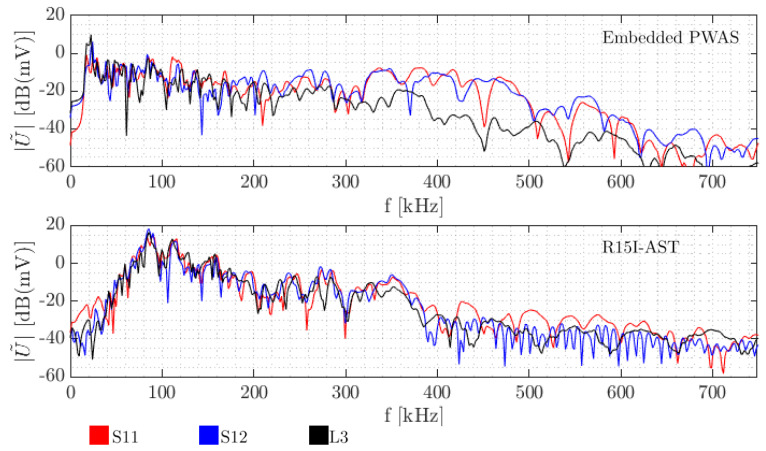
Typical amplitude spectrum of Hsu-Nielsen sources as recorded by the small and large embedded PWAS and the surface-mounted sensor.

In order to compare the sensitivity of the different piezoelectric sensors in use in the experiment, Hsu-Nielsen tests with the procedure described in Section 2.1 were performed. Results of specimens S11 and S12, with small (7 mm diameter) piezoelectric sensors, and L5, with a larger (20 mm diameter) piezoelectric sensor embedded were analysed. Amplitude spectra are shown in Figure 5.

When comparing the embedded sensors, it stands out that up to 300 kHz, results are fairly similar. Beyond 380 kHz, sensitivity of the larger sensor declines. This can be partly attributed to an aperture effect, as the sensor integrates displacement over the contact area. For wavelengths that are increasingly smaller than the sensor diameter, the full wavelengths enclosed by the contact area increasingly cancel out.

For the same experiments (under the same conditions), this decreasing trend is also visible in the R15I-AST sensors from 350 kHz. Furthermore, the embedded sensors seem to be more sensitive than the R15I-AST to lower frequencies (<80 kHz), which can be partly attributed to the heavier reliance of the latter on resonant behaviour.

#### 3.1.2. Embedded Sensor Performance during Four-Point Bending Experiments

**Figure 6:**
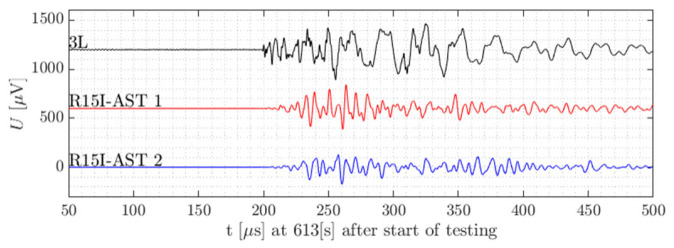
A single AE hit as measured by the embedded sensor L3 and the two R15I-AST sensors [36].

During the experiments outlined in Section 2.1 AE waveforms were acquired by both the embedded sensors and the surface-mounted R15I-asts. This is exemplified in Figure 6.

**Figure 7:**
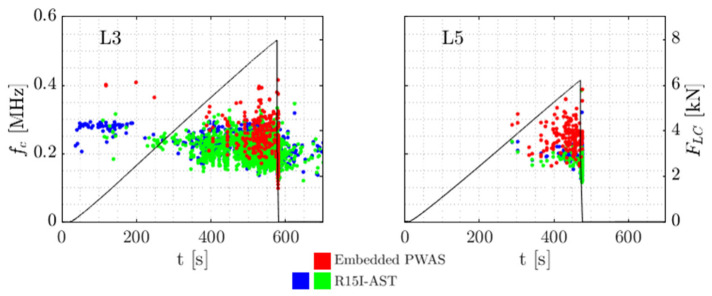
Centroid frequency $f_{c}$ of all measured signals over time and load cell force $F_{LC}$ for the large ‘L’ embedded sensors and the two R15I-ASTs. Note L3 has been subject to noise removal as is explained in Section 3.1.3. The final outcome is shown here.

**Figure 8:**
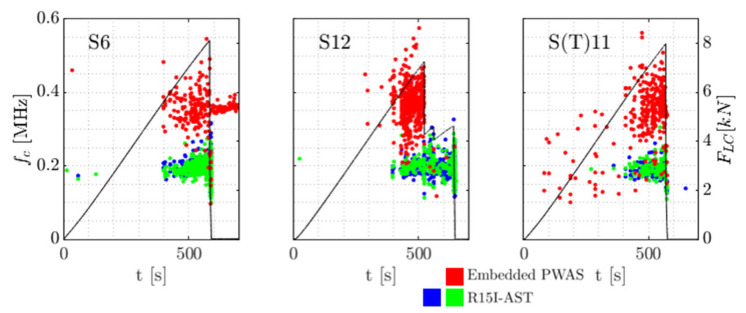
Centroid frequency $f_{c}$ of all measured signals over time and load cell force $F_{LC}$ for the small ‘S’ and ‘ST’ embedded sensors and the two R15I-ASTs.

For the specimens tested, it stands out the embedded sensor is able to receive AE signals up to failure, either when loaded under compression or in tension. This may be seen in Figure 7 and Figure 8. The frequency content acquired in the destructive tests seems generally higher than the frequencies in the preliminary tests with Hsu-Nielsen, possibly due to the different spectrum of the damage-induced source signals. The larger embedded sensor recorded AE signals with centroid frequencies mostly between 200 kHz and 300 kHz. For the smaller embedded sensor, this range was pronounced between 350 kHz and 450 kHz. For the R15I-AST sensors on all specimens, centroid frequencies tend to be between 150 kHz and 350 kHz, conforming to the transfer function of the sensor [40].

#### 3.1.3. Embedded Sensor Noise Mitigation during Four-Point Bending Experiments

**Figure 9:**
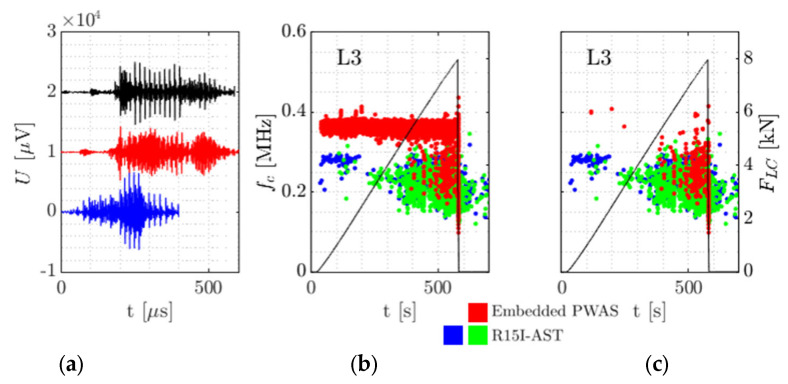
Noise mitigation on specimen L3: (**a**) Three waveforms measured in the reference timeslot between 162 s and 242 s after the start of test. They are translated in time and voltage to fit in the same image. The dissimilarity between the waveforms is between 0.65 and 0.79. (**b**) Centroid frequency *f_c_* of all measured signals over time and load cell force $F_{LC}$ for the embedded sensor and the two R15I-ASTs. (**c**) Equivalent to (**b**), but with noise removed.

In the first stage of testing it was noted that the embedded sensor tended to pick up extensive amounts of continuous noise. Based on the preliminary tests from Section 3.1.1, this was not expected. In one case, specimen L3, no continuous-type noise but burst-type signals were acquired. A large amount were amassed continuously from the start of the testing. In Figure 9a three waveforms measured by the embedded sensor in early stage of testing are shown. Next to that the accumulation of signals, represented by their centroid frequency $f_{c}$, over time is illustrated.

Due to their early and repeating appearance (burst repetition frequency around 50 kHz), in the wave forms, these signals are considered noise. In a second batch of experiments it was identified that the continuous noise related to a metal-to-CFRP contact in the four-point bending setup. After insulating the aluminium loading tabs with polyester film tape, no such behaviour occurred anymore. Therefore this effect is not directly attributed to possible triboelectricity effects due to the external wiring [41]. Further detailed investigation of triboelectricity effects will be considered in the future research.

Measures were taken to prevent the noise from interfering with the analysis. In assessing the performance and features of the embedded AE sensor, a waveform similarity approach as described in Section 2.2 is utilised to distinguish signals of interest from noise.

**Figure 10:**
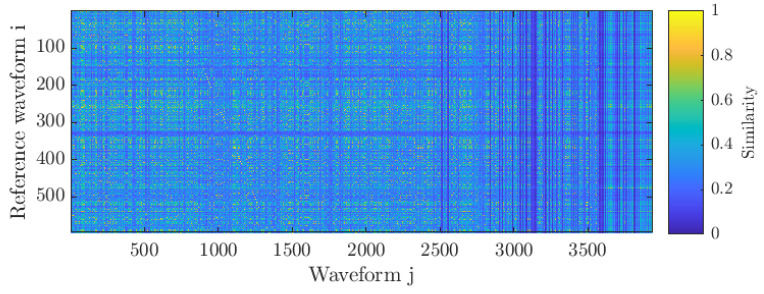
Similarity matrix with reference ‘noise’ waveforms correlated with the rest of data. Note the faint diagonal line between j = 800 to j = 1300, indicating autocorrelation.

For specimen L3, a total of 598 waveforms measured by the embedded sensor between 162 s and 242 s after the start of loading were used as a reference and compared to all 3939 waveforms from the embedded sensor. The resulting similarity matrix with the waveforms in chronological order is shown in Figure 10.

The figure shows that waveforms j (sorted in chronological order) became increasingly dissimilar from the reference waveforms (up to j = 2500 this was not the case). Regarding reference waveforms i, the different lighter and darker shades imply variations within the reference waveforms. As such, deviating waveforms may contain useful information. The criterion for defining noise was defined such that the waveform under investigation should be akin to at least 5 of the reference waveforms. In this context, ‘akin’ implies a dissimilarity of at most 0.65.

In Figure 9 the waveforms shown on the left have a dissimilarity between 0.65 and 0.79, visualising the boundaries of the criterion. In the middle and right, the effect of removing the noise signals may be seen. Although waveforms similarity inherently takes into account similarity in frequency content, the comparison shows the noise mitigation is sensitive enough to retain dissimilar signals with a similar frequency content, as exemplified by the waveforms in the 350–400 kHz band.

In the identification of waveforms related to embedded sensor-induced damage, measurements from the embedded sensor were not included in the analysis to prevent possible influence of damage to the sensor during the experiments. Only waveforms measured near-simultaneously (within 10 μs) by the two surface-mounted R15I-ASTs were examined.

### 3.2. Identification of Damage Mechanisms Occurring Due to the Embedding of a Piezoelectric Sensor

#### 3.2.1. Visual Assessment of Damage Mechanisms

**Figure 11:**
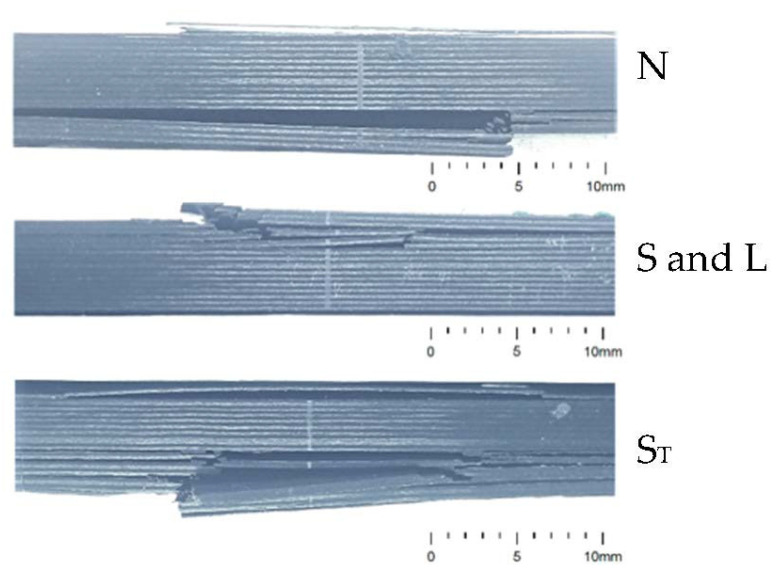
A side view showing damage occurring in different types of specimens [36].

During the four-point bending experiment, a number of damage mechanisms were registered. In general from $F_{Lc}$ = 6 kN and beyond the upper laminae between the loading tabs showed signs of damage, in the form of delamination and fibre fracture on the top surface. Failed specimens are depicted in Figure 11 [38].

For baseline specimens that had no sensor embedded (N) this damage on the compressive side is protracted up to near the fourth lamina before gross failing in the tension side, including delamination and fibre breakage in three of the specimens. In one of the specimens of type N, failure occurred solely on the compressive side close to the loading tabs. For specimens failing on the tension side, this location was between 8 mm and 21 mm from the centre of the specimen.

In specimens where a sensor was embedded on the compressive side (S and L type specimens) of the beam final failure occurred on the compressive side in an instantaneous manner reaching from the top surface to the laminae that contained the embedded sensor. Generally the location of rupture seemed to coincide with the interface between the CFRP and the embedded sensor. In one specimen however, failure was near the centre. The rupture can be considered as a combination between delamination and compressive fibre failure. Furthermore, the sensors did fail together with the GFRP insulation. This can be regarded as an extra damage mechanism, due to a combined in-plane compression and out-of plane shear stress (related to the bending setup).

Like in the baseline specimens, for the specimens with sensors embedded on the tension side of the beam (S_T_) the initial damage on the compressive side did not extend beyond the top laminae. At increasing loads, acute failure occurred on the tension side of the specimens, approximately 4.5 mm to 8 mm off-centre up to the laminae with embedded sensors. Similar to the s and L specimens, degradation modes relate to delamination and tensile fibre failure. Failure of the sensor itself and insulation is caused by combined in-plane tension and out-of plane shear stress.

In short, the different damage mechanisms are given in Table 1.

#### 3.2.2. Assessment of Energy and Cumulative Energy

**Figure 12:**
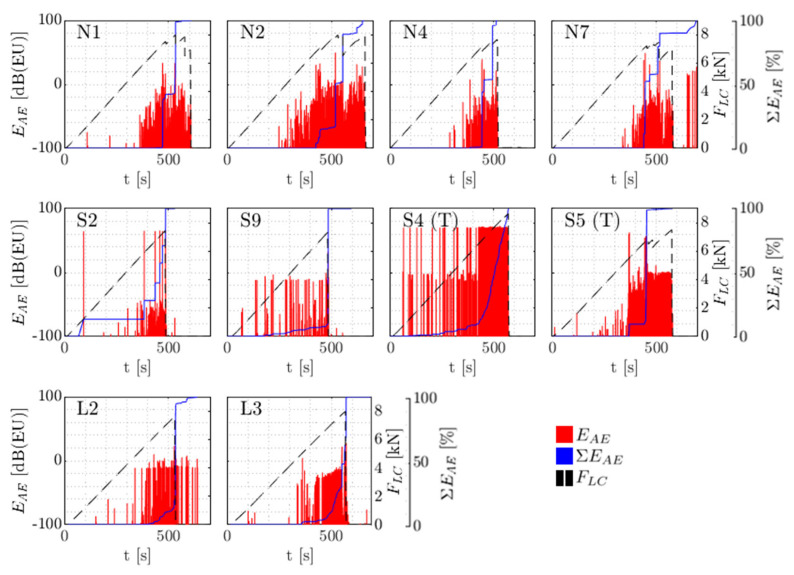
Energy per hit $E_{AE}$ and cumulative energy [% of total] over time and crosshead force $F_{LC}$.

During the failure of specimens as described in Section 3.2.1 two R15I-AST surface-mounted transducers were continuously recording AE. Next to the registration of the waveforms, features such as waveform energy $E_{AE}$ were directly extracted. As stated in Section 3.1.3, to prevent the background noise contaminating the assessment, only AE recorded simultaneously (first threshold crossings within 10 μs from each other) by both sensors is considered. Waveform energy $\;(E_{AE}$) and cumulative energy $(\Sigma E_{AE}$) over time are shown for the different specimens in Figure 12.

From the figure it can be noted that in baseline (N) specimens, energy-rich AE started to occur from 6000 N onwards and increased in energy around the failure load of the specimens. For specimens with large embedded sensors on the compression side of the specimen (L2 and L3), energy content seems to be fairly comparable to the baseline specimens. For their counterparts with smaller PWAS, AE in specimens S2 and S9 seem to commence relatively early, around 1–1.5 kN. For specimens with embedded PWAS on the tensile side (S_T_4 and S_T_5) it appears that the recorded energy is larger than with the other specimens throughout the testing. This can be seen in Table 2 as well, with the cumulative energy of S_T_ specimens being higher than in other specimens (with reasonable statistically significance). When considering specimen S_T_5, high-energy AE around 5000 kN is observed, followed by lower-energy AE. This reminisces of the observations from Xiao et al. [26]. The lower-energy AE after 5000 kN however is not generally lower than experienced in baseline specimens.

#### 3.2.3. Identification of Damage Mechanisms Using Waveform Similarity

Building further on the measurement of AE as explained in Section 2.1, effort is made to identify AE that is characteristic to failure related to the embedding of a piezoelectric sensor. Interpretation relies on the comparison between clusters of AE measured in the sensor-less baseline specimens, and those from the specimens with sensors embedded.

**Figure 13:**
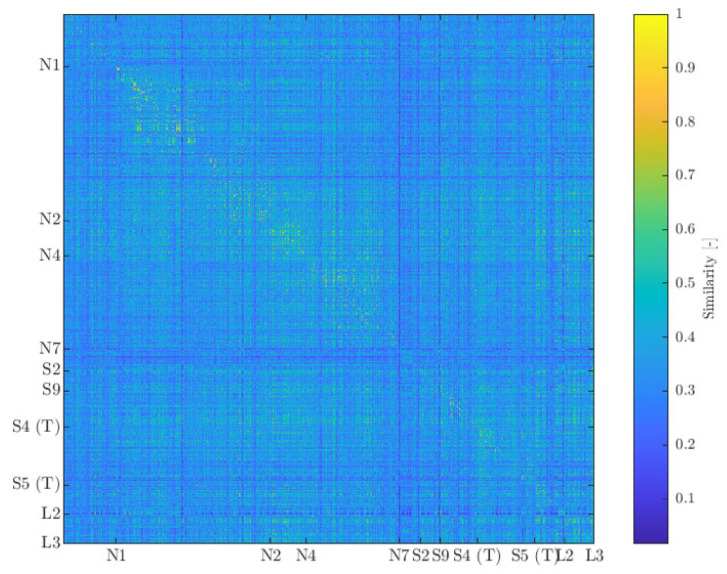
Similarity matrix containing all 15,081 waveforms measured during loading and failure of the ten specimens. Indices relate to the last wave form from the specimen.

Using the method described in Section 2.2, a similarity matrix is constructed, containing similarity information of AE waveforms captured during the loading and failure of all specimens. The obtained similarity matrix can be seen in Figure 13. In this figure the waveforms are ordered chronologically. Near the diagonal in Figure 13, lighter colours with a similarity between 0.8 to 1 are visible. This suggests that similar waveforms are grouped in time and may be attributed to similar damage mechanisms in the same specimen. Further from the diagonal similarity values overall seem to be somewhat lower, but the extent thereof is mostly waveform-specific. Also there are recurring patterns of higher similarity as well as waveforms that do not tend to relate to the others.

**Figure 14:**
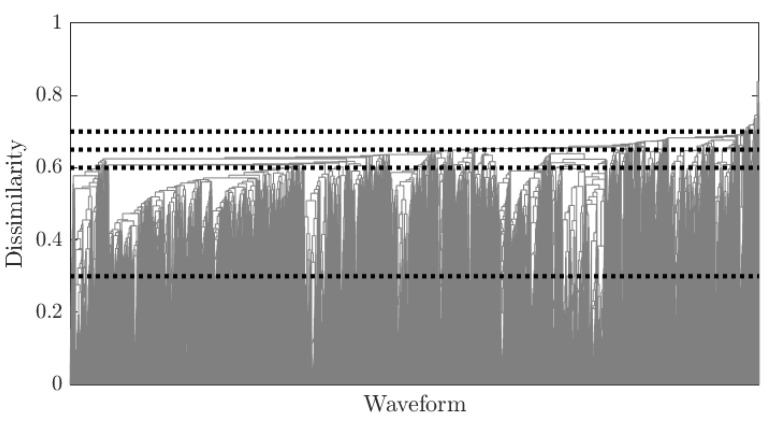
Dendrogram linking the data from the similarity matrix in Figure 13. The dashed lines represent the thresholds considered in the definition of clusters. Note that along the horizontal axis the waveforms are no longer ordered sequentially.

To further elaborate on above observations and in the pursuit of clustering the waveforms, a dendrogram (Figure 14) is formed using the dissimilarity as explained in Section 2.2. It is noticeable from the dendrogram that there are few groups of waveforms that have a high mutual similarity (or low dissimilarity, <0.3) while the bulk of waveforms tend to distinguish themselves in the 0.3–0.7 dissimilarity range.

For the purpose of clustering a similarity threshold is defined. This threshold separates waveforms along a maximum value of dissimilarity. The value for this threshold is not trivial: a low dissimilarity threshold can result in highly similar clusters, but those may be excessively specific to individual specimens and not relate to global phenomena across multiple specimens. On the other hand, a high dissimilarity threshold may overlook relevant clusters. Variations in local FRP fibre volume fraction and direction, the initiation of damage, the placement of the sensor and the thickness of the couplant can cause variations in wave propagation details between different specimens. This results in AE from similar damage that may have relatively high levels of dissimilarity. To ensure variations between specimens (of the same type) do not influence the identification process, the threshold is selected such that major clusters contain waveforms from multiple specimens of the same type. It should be noted this selection relies on the assumption that the dissimilarity between different damage mechanisms is larger than the dissimilarity between multiple specimens given the same damage mechanism.

**Figure 15:**
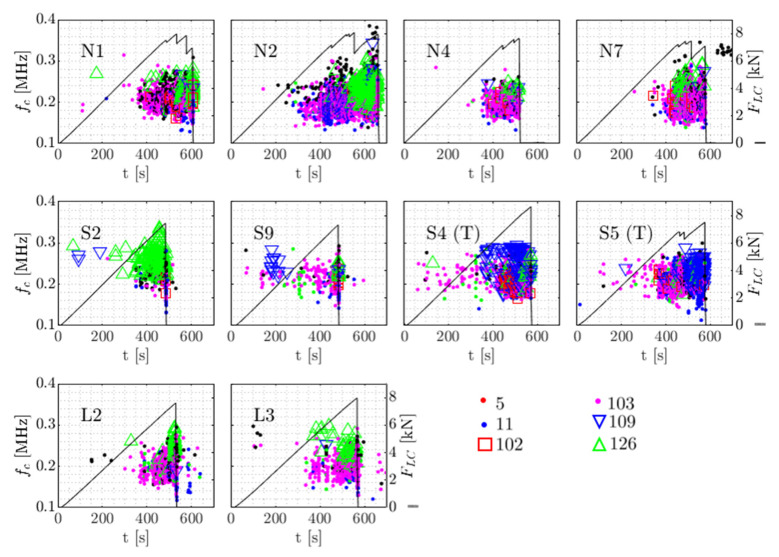
The AE clusters for each specimen, centroid frequency $f_{c}$ of the waveforms in the clusters and the applied load $F_{LC}$.

**Figure 16:**
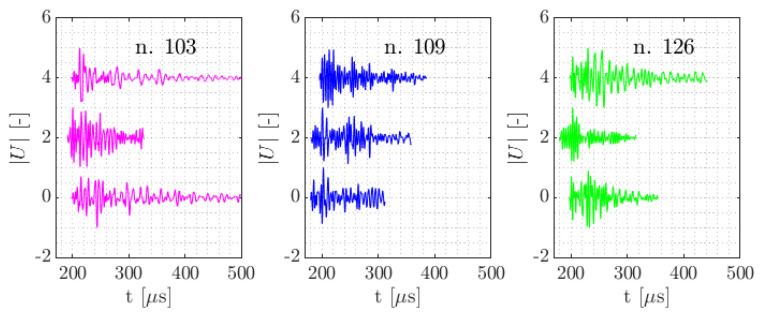
Normalised examples of waveforms from cluster numbers 103, 109 and 126.

For a dissimilarity threshold of 0.65, in total 154 clusters were formed, with the bulk of waveforms (92%) found within 8 clusters. Table 3 describes the distribution of clusters over the specimen types. In Figure 15, the clusters are visualised as centroid frequency over time. In Figure 16 three normalised waveforms are shown belonging to clusters 103, 109 and 126.

From the table and Figure 15 L specimens appear to show behaviour most alike to baseline N specimens, with a comparable distribution of clusters in number and over testing time. Note that this can be mirrored to the failure load of the L specimens, which compared to S specimens is relatively similar to N specimens. AE from S specimens are slightly less represented by the proposed clusters (77% of all waveforms) with the remaining waveforms being placed in smaller clusters with high reciprocal dissimilarity. Cluster 126, with a relatively high centroid frequency, occurs in S2 from around $F_{LC}$ = 2 kN whereas in baseline (N) specimens, this cluster is far more limited in number and associated with final failure. In S_T_ specimens most distinct is the abundance of cluster 109, including 325 waveforms uniformly divided over the two specimens. In the other specimens the occurrence is almost negligible (40 waveforms in all other specimens combined). The high repeatability of the cluster possibly allows attribution to damage mechanisms related to the specific embedding procedure and the combined in-plane tension stress and out-of-plane shear stress that is experienced in S_T_.

**Figure 17:**
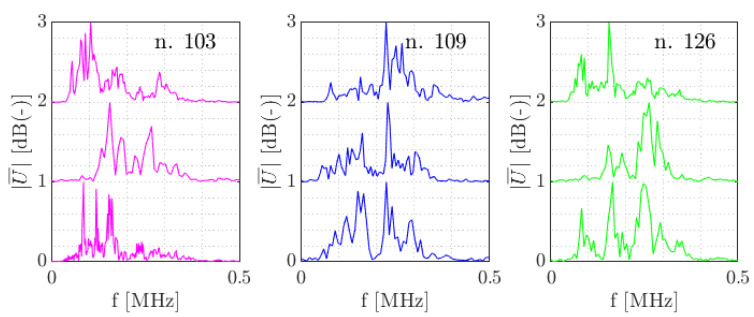
Normalised amplitude spectra belonging to the waveforms from Figure 16.

In Figure 16 and Figure 17 similarity of the waveforms within clusters and between clusters can be visually assessed. In these figures the waveforms per cluster are coming from different specimens and are picked randomly. In the time domain, it may be seen there are similarities around the onset of some of the waveforms per cluster. In frequency domain it appears that cluster 103 has most frequency content below 200 kHz. In cluster 109 the low frequency content around 200 kHz and a large frequency content around 220 kHz show to be the uniting factors. In cluster 126 such relations are less apparent.

To summarise, clusters are formed using waveform dissimilarity. This clustering generally represents AE behaviour that is repeatable over different specimens with the same embedded sensor type. Furthermore AE cluster distribution in specimens with embedded sensor is compared to that in the baseline specimens. It may be noted that specimens with a large (L type, 20 mm diameter) sensor embedded on the compressive side of the bending setup exhibit comparable AE behaviour to that of the baseline specimens. Conversely in specimens with smaller sensors embedded on the same side (S type, 7 mm diameter) AE tends to be relatively dissimilar in general, but clusters with high frequency content seem to occur earlier than in baseline specimens. For specimens with the embedded sensor loaded in tension (S_T_, 7 mm diameter), a cluster (cluster 126) is noted that appears to be specific to this type of specimen. This suggests the presence of a possible damage mechanism related to the embedded sensor experiencing combined in-plane tension stress and out-of-plane shear stress.

## 4. Discussion

### 4.1. Measuring AE with Embedded Sensors

Acoustic emissions originating from mechanical degradation sources were measured in CFRP specimens with embedded PWAS and standard surface-mounted piezoelectric sensors. Two sizes of embedded PWAS (20 mm and 7 mm diameter) were compared in terms of performance and sensitivity. Typically, embedded PWAS were able to acquire AE up to final failure. In the measurement of damage-related AE from a flexural strength test, the smaller embedded PWAS (7 mm diameter) recorded AE with a centroid frequency typically ranging between 350 kHz and 450 kHz. For the larger embedded PWAS (20 mm diameter) centroid frequencies were determined to be generally between 200 kHz and 300 kHz. In all cases, the reference R15I-AST sensors measured centroid frequencies between 150 kHz and 350 kHz. The variation in sensitivity between the larger and smaller embedded PWAS is partly attributed to an aperture effect by taking into account the wavelength of the propagating wave of interest. This implies that embedded PWAS dimensions and FRP wave propagation properties should be carefully assessed in the design and analysis of an FRP structure with embedded PWAS.

### 4.2. Waveform Similarity Assessment for Identification of Noise and Damage Mechanisms

A waveform similarity approach was used for defining AE clusters and in the processing of noise. It was shown using average linkage and a dissimilarity threshold that clusters of AE can be formed that are overarching different specimens while being specific enough to accentuate the effects found in different specimen types. This confirms the assertion that the dissimilarity of AE between different types of specimens is larger than the dissimilarity between multiple same-type specimens. As is seen in Figure 14, Figure 16 and Figure 17 differences in dissimilarity within clusters are relatively large while between the clusters the differences in dissimilarity are relatively low. This indicates that for a small difference in dissimilarity threshold, large differences in the formation of clusters are obtained. Cluster 109, that is assigned to be specific to specimens with embedded PWAS on the tensile side of the bending test, is considered of note due to its occurrence being almost exclusive to this type of specimens as well as its distribution being comparable over the two specimens of this type.

### 4.3. Identification of Damage Mechanisms Occurring Due to the Embedding of a Piezoelectric Sensor

From the combination of waveform similarity and AE energy assessment it may be concluded that the AE behaviour of the two specimens with embedded PWAS on the tensile side of the bending load are distinctly different from the baseline and other specimens. The identified AE phenomena are envisioned to be associated to a tensile cohesion-related failure, since the strength of the GFRP-resin-CFRP interface is subject to large uncertainty. Tensile fracture of the PWAS is not expected as a likely source given the low stiffness of GFRP and resin insulation, as confirmed by the embedded PWAS in specimen S_T_11 that recorded up to gross failure. Given the inaccessibility of the location of damage for detailed inspection during the test, further validation related to the degradation mode is needed.

Between baseline specimens and specimens with embedded PWAS on the compressive side under the bending load, no clear relation in terms of AE can be established. Although visually the damage mechanisms in these specimens are different compared to baseline specimens, this did not translate to sufficiently consistent trends in AE.

### 4.4. Outlook and Future Research

From a wider perspective, the ability to relate AE to embedded PWAS-affiliated and non-affiliated degradation mechanisms allows for the monitoring of a structure with embedded PWAS with respect to the health of the structure. Further, the monitoring for embedded PWAS-induced degradation facilitates the development of design criteria for structures with embedded PWAS. In this regard, the observation that AE from PWAS-induced damage is distinguishable from other sources, prior to specimen gross failure and depending on loading direction, is of value. It is considered that this work will also pave the way for more widespread use of embedded PWAS, such as in larger-scale FRP structures with embedded arrays or distributed configurations.

This research can be seen as a preliminary step towards assessing the influence of embedded PWAS size and location on their measurement capabilities and on the structural integrity of the host structure. As such, it is acknowledged that the topics and outcomes presented in this research can be elaborated upon, by means of extended experiments and numerical simulations. Measuring different wave modes with different wave lengths and the effect on the sensitivity of embedded PWAS deserves further research. Evaluation with respect to the sensitivity of the embedded PWAS is envisioned on different host materials, such as GFRP and sandwich structures. The clustering method used may be compared to other clustering procedures that are used typically in the classification of FRP damage mechanisms. In this context, the respective qualities of the formed clusters may be assessed. Moreover, it is recognised that increasing the population size of the specimens will help drawing more accurate and definite conclusions on the formation of AE clusters related to embedded PWAS-induced damage. Also, this will allow specimens in the destructive testing to be stopped prematurely so that they can be inspected microscopically for the initiation of damage mechanisms. The premature inspection will facilitate attributing clusters to damage mechanisms. In a further stage, a database of clusters of waveforms with identified damage mechanisms may be used to quantify the nature and extent of damage in similar structures. The features of AE signals from identified damage mechanisms can also be used to develop possible damage indices to better estimate the remaining load bearing capacity or remaining lifetime.

## 5. Conclusions

Using data from destructive four-point bending tests performed on thick CFRP specimens with embedded PWAS the performance of the PWAS in terms of endurance and spectral sensitivity for AE measurement was investigated. Furthermore, a waveform similarity assessment method was employed to identify clusters of AE signals that relate to embedded PWAS-induced failure mechanisms. It was found that:

(1) The size of the embedded PWAS has an effect on the frequency content of the measurement of AE signals; Damage-related AE signals in CFRP specimens were measured with a centroid frequency between 350 kHz and 450 kHz by the 7 mm diameter embedded PWAS, while for the 20 mm embedded PWAS the range was between 200 kHz and 300 kHz. For comparison, no change in frequency content in the AE signals was measured by a reference surface-mounted sensor for the different specimens.
(2) Damage mechanisms in the CFRP laminates due to embedded PWAS when placed in the tension side in the bending experiment exhibited AE that were reasonably distinguishable from other sources of AE. This was confirmed when assessing waveform similarity and AE energy in specimens with and without embedded sensors. When loaded under compression, no major additional degradation AE due to the embedment process was identified.

## Figures and Tables

**Table 1:** Damage mechanisms as registered during four-point bending. ‘v’,’-/v’ and ‘-’ signify that the mechanism is found in the bulk of specimens, in a single specimen or not at all, respectively.

Damage Mechanism	N	S	S_T_	L
Preliminary delamination on compressive side	v	v	v	v
Preliminary fibre breakage on compressive side	v	v	v	v
Extensive fibre breakage on compressive side	-/v	v	-	v
Extensive fibre breakage on tensile side	v	-	v	-
Sensor system failure on compressive side	-	v	-	v
Sensor system failure on tensile side	-	-	v	-

**Table 2:** Cumulative energy $\Sigma E_{AE}$.

	N	S	S_T_	L
$\Sigma E_{AE}$ [$\times 10^{6}$EU]	4.0 × 10^2^	1.3 × 10^4^	1.1 × 10^6^	3.1 × 10^2^
	9.0 × 10^2^	1.2 × 10^2^	2.5 × 10^4^	8.1 × 10^1^
	3.5 × 10^2^			
	7.0 × 10^2^			

**Table 3:** Major clusters formed using a threshold of 0.65. For each type of specimen, the percentage of waveforms caught in the clusters is given.

Cluster Number	N [%]	S [%]	S_T_ [%]	L [%]
5	3	3	3	5
11	19	12	13	8
21	2	0	3	1
102	0	1	4	0
103	61	37	53	70
104	3	4	0	1
109	0	1	12	0
126	4	19	1	6
Total	92	77	89	91

## Data Availability

The data presented in this study are available upon request from the corresponding author.
